# P-1676. Utilization of a Syndromic Gastrointestinal Panel in Patients Hospitalized >3 Days in a Quaternary Care Academic Medical Center

**DOI:** 10.1093/ofid/ofaf695.1850

**Published:** 2026-01-11

**Authors:** Kathleen McKinley, Louise Lie, Amanda Harrington

**Affiliations:** Loyola University Medical Center, Maywood, Illinois; Loyola University Medical Center, Maywood, Illinois; Loyola University Chicago, Maywood, IL

## Abstract

**Background:**

Both American College of Gastroenterology (ACG) 2016 Clinical Guidelines and IDSA/ASM 2024 Guide to Utilization of the Microbiology Laboratory for Diagnosis of Infectious Diseases have recommendations for utilization of diagnostic studies in acute gastroenteritis. Indications include cases of dysentery, moderate-to-severe disease, nosocomial, or persistent diarrheal illnesses lasting >7 days or immunocompromised status. General practice in many clinical microbiology laboratories is that routine enteric pathogen testing (other than for C. difficile) is often restricted for patients who have been hospitalized >3 days due to low clinical yield. Larger syndromic panels are an effective analytical tool for identification of multiple etiologies from one sample type. It is less well understood how clinically effective the utilization of these panel are in hospitalized patients.

**Methods:**

We reviewed the utilization and results from the BioFire FilmArray Gastrointestinal Panel (GIP) ordered for patients after >3 days of hospitalization from January 2018 to March 2024 at a Quarternary Care Academic Medical Center.

**Results:**

465 tests were ordered for patients after >3 days of hospitalization during this period. Although the overall percentage of test orders were low and overall utilization steadily decreased, we saw an increase in ordering for this patient population from 0.8% in 2018 to 6.1% in 2024. Excluding the C. difficile target, only 56/465 (12%) had a target detected. When EPEC, EAEC, and ETEC were also excluded, only 44/465 (8.6%) had a target detected. Norovirus (22) was the most common target detected, followed by Sapovirus (6). No other target had more than 2 detections. Limited chart review demonstrated although the majority of cases have consulted the infectious disease (ID) team, GIP is consistently ordered outside of guideline indications or without ID recommendations.

**Conclusion:**

These data support the best practice recommendations that testing for routine enteric pathogen testing is of low clinical utility in patients who have been hospitalized >3 days. Additional decision support for utilization within the current recommended indications would likely improve overall utilization. A stand-alone assay for Norovirus may be a more effective diagnostic option.

**Disclosures:**

Kathleen McKinley, MT(ASCP), Beckman Coulter: Grant/Research Support|bioMerieux/BioFire: Grant/Research Support Amanda Harrington, PhD, Beckman Coulter: Grant/Research Support|bioMerieux/BioFire: Advisor/Consultant|bioMerieux/BioFire: Grant/Research Support|bioMerieux/BioFire: Honoraria|BioRad: Advisor/Consultant|Selux: Grant/Research Support

